# Radiation-related quality of life parameters after targeted intraoperative radiotherapy versus whole breast radiotherapy in patients with breast cancer: results from the randomized phase III trial TARGIT-A

**DOI:** 10.1186/1748-717X-8-9

**Published:** 2013-01-07

**Authors:** Grit Welzel, Angela Boch, Elena Sperk, Frank Hofmann, Uta Kraus-Tiefenbacher, Axel Gerhardt, Marc Suetterlin, Frederik Wenz

**Affiliations:** 1 Department of Radiation Oncology, University Medical Centre Mannheim, University of Heidelberg, Mannheim, 68167, Germany; 2 Department of Gynecology, University Medical Centre Mannheim, University of Heidelberg, Mannheim, 68167, Germany

**Keywords:** Quality of life, Targeted intraoperative radiotherapy, Breast cancer, Boost, TARGIT-A trial

## Abstract

**Background:**

Intraoperative radiotherapy (IORT) is a new treatment approach for early stage breast cancer. This study reports on the effects of IORT on radiation-related quality of life (QoL) parameters.

**Methods:**

Two hundred and thirty women with stage I-III breast cancer (age, 31 to 84 years) were entered into the study. A single-center subgroup of 87 women from the two arms of the randomized phase III trial TARGIT-A (*TARG*eted *I*ntra-operative radio*T*herapy versus whole breast radiotherapy for breast cancer) was analyzed. Furthermore, results were compared to non-randomized control groups: n = 90 receiving IORT as a tumor bed boost followed by external beam whole breast radiotherapy (EBRT) outside of TARGIT-A (IORT-boost), and n = 53 treated with EBRT followed by an external-beam boost (EBRT-boost). QoL was collected using the European Organization for Research and Treatment of Cancer Quality of Life Questionnaires C30 (QLQ-C30) and BR23 (QLQ-BR23). The mean follow-up period in the TARGIT-A groups was 32 versus 39 months in the non-randomized control groups.

**Results:**

Patients receiving IORT alone reported less general pain (21.3 points), breast (7.0 points) and arm (15.1 points) symptoms, and better role functioning (78.7 points) as patients receiving EBRT (40.9; 19.0; 32.8; and 60.5 points, respectively, *P* < 0.01). Patients receiving IORT alone also had fewer breast symptoms than TARGIT-A patients receiving IORT followed by EBRT for high risk features on final pathology (IORT-EBRT; 7.0 versus 29.7 points, *P* < 0.01). There were no significant differences between TARGIT-A patients receiving IORT-EBRT compared to non-randomized IORT-boost or EBRT-boost patients and patients receiving EBRT without a boost.

**Conclusions:**

In the randomized setting, important radiation-related QoL parameters after IORT were superior to EBRT. Non-randomized comparisons showed equivalent parameters in the IORT-EBRT group and the control groups.

## Background

Intraoperative radiotherapy (IORT) is a new treatment option for women with early stage operable breast cancer, and it is increasingly used in clinical practice. In 2000, the international randomized phase III trial TARGIT-A (NCT00983684; *TARG*eted *I*ntra-operative radio*T*herapy versus whole breast radiotherapy for breast cancer) was started to investigate the non-inferiority of targeted intraoperative radiotherapy (IORT) given in a single dose to the tumour bed during breast-conserving surgery (BCS) as compared to conventional external beam whole breast radiotherapy (EBRT) in early stage breast cancer. In the presence of risk factors identified in the final pathology (invasive lobular carcinoma, extensive intraductal component, involved margins, lymphovascular invasion) postoperative EBRT is added after IORT per protocol (IORT-EBRT). The first analysis of the trial, published in June 2010, showed a 4 year local recurrence rate of about 1% and a clinically relevant toxicity rate of about 3% in both arms [1,2].

The primary aim of the present analysis was to assess radiation-related QoL parameters in the first 123 women from a single center participating in the TARGIT-A trial. The secondary aim was to compare TARGIT-A IORT-EBRT patients with two non-randomized control groups of patients treated with *(i)* IORT as a tumor bed boost followed by EBRT outside of TARGIT-A (IORT-boost) or *(ii)* EBRT followed by an external-beam boost to the tumor bed (EBRT-boost). The primary end points were the global health status, restrictions in daily activities (role functioning), and general pain subscales from the European Organization for Research and Treatment of Cancer (EORTC) Quality of Life Questionnaire C30 (QLQ-C30, version 3) [3], and the breast symptoms and arm symptoms subscales from the EORTC Quality of Life Breast Cancer Module (QLQ-BR23) [4].

## Methods

### Treatment and patients

This study was a single center cross-sectional analysis. To qualify for the analysis, patients had to be randomized in the TARGIT-A trial between 2002 and 2009 from the University Medical Centre Mannheim. The full protocol of TARGIT-A is available online [5].

All eligible TARGIT-A patients were treated as follows Arm A: IORT with 50 kV X-rays (INTRABEAM™ system, Carl Zeiss Surgical, Oberkochen, Germany) delivering 20 Gy at the applicator surface during BCS (IORT group). In the presence of risk factors this was followed by EBRT with 46 Gy in 23 fractions or 50 Gy in 25 fractions to the whole breast (IORT-EBRT group). Arm B: EBRT with 56 Gy in 28 fractions to the whole breast postoperatively without boost (EBRT group). The rationale of the EBRT treatment was based on the German breast cancer study group (GBSG) recommendations at the time of study initiation.

The patients of the control groups outside of TARGIT-A were treated between 2002 and 2006 at the same centre. Patients received BCS and either a combination of 20 Gy IORT and postoperative EBRT of 46 Gy in 23 fractions (IORT-boost group) or postoperative EBRT of 50 Gy in 25 fractions followed by an external-beam boost to the tumor bed of 16 Gy in 8 fractions (EBRT-boost group). EBRT was initiated after completion of wound healing and/or chemotherapy. All patients received systemic therapy according to the St. Gallen consensus recommendations. Chemotherapy was routinely given before EBRT. Endocrine therapy was started 8–14 days after surgery or after completion of chemotherapy.

From June 2002 to February 2009, 88 patients (72%) out of 123 patients accrued to the TARGIT-A trial at our center consented to participate in the QoL study. Forty-six and 42 patients were allocated in the targeted intraoperative radiotherapy (Arm A, IORT +/− EBRT) and external beam radiotherapy (Arm B, EBRT) groups, respectively. Sixteen patients allocated to intraoperative radiotherapy received intraoperative radiotherapy plus external beam radiotherapy postoperatively (IORT-EBRT group). Five patients did not receive IORT: Four patients were treated with 56 Gy external beam radiotherapy postoperatively, one patient refused external beam radiotherapy. All patients received treatment as mentioned above. The patients in Arm B were treated with 28 x 2 Gy to the whole breast, and none of them received an additional boost. The mean age at the TARGIT-A entry was 64.7 years (median 65 years, range, 47 to 84), the mean follow-up time was 32.1 months (median 25 months, range, 9 to 94).

In addition, this single-center subgroup of patients from the TARGIT-A trial was compared to patients of our center treated with IORT as a tumor bed boost followed by EBRT outside of TARGIT-A (IORT-boost group), and patients treated with EBRT followed by an external-beam boost to the tumor bed (EBRT-boost group). All patients treated between 2002 and 2006 with 20 Gy IORT as a tumor bed boost followed by 46/2 Gy EBRT to the whole breast outside of TARGIT-A were asked to participate in the IORT-boost control group. 90 patients (96%) out of 94 patients consented to participate. All patients treated with 50/2 Gy EBRT to the whole breast followed by 16/2 Gy EBRT-boost to the tumor bed between 2005 and 2006 were asked to participate in the EBRT-boost group, and 53 patients (85%) out of 62 patients participated. For these groups, the mean age at the time of surgery was 56.3 years (median 56 years, range, 31 to 81), and the follow-up time was 39.1 months (median 41 months, range, 8 to 64).

Most patients suffered from breast cancer stage I or stage II. One patient (2%) in the EBRT group, 4 patients (4%) in the IORT-boost group and 11 patients (21%) in the EBRT-boost group had breast cancer stage III. At the time of survey, all patients were disease free. Further patients’ characteristics are summarized in Table 1. We compared the patients that participated in the QoL study with those who declined participation to assess for sample bias, and there were no differences regarding demographic and clinical variables (data not shown). Also compared with patients in the whole TARGIT A trial [1], TARGIT-A patients in our study had largely similar demographic and clinical characteristics.

**Table 1:** Patient characteristics (as treated)

	**TARGIT-A**							**EBRT+boost**				
	**IORT (n = 25)**		**EBRT (n = 46)**		**IORT-EBRT (n = 16)**			**IORT-boost (n=90)**		**EBRT-boost (n=53)**		
	**No.**	**%**	**No.**	**%**	**No.**	**%**	***P*** *	**No.**	**%**	**No.**	**%**	***P*** **
Age, years												
Mean	65.5		65.2		61.8		0.276	60.1		49.9		<0.001
SD	8.5		8.2		6.0			11.1		9.2		
Married/partnered	14	58	22	48	13	81	0.066	58	64	39	76	0.191
Employed	4	16	9	20	0	0	0.185	27	31	31	60	<0.001
Months since BCS												
Mean	32.7		30.6		35.6		0.641	34.2		47.5		<0.001
SD	19.1		17.2		19.6			13.7		7.4		
Tumor size												
0-1 cm	8	32	14	30	3	19	0.376	13	14	6	11	0.414
1-2 cm	16	64	25	34	9	56		49	54	23	43	
> 2 cm	1	4	7	15	4	25		28	31	24	45	
Nodal involvement												
N0	23	92	36	78	14	88	0.622	66	73	34	65^a^	0.253
N1	2	9	9	20	2	12		20	22	11	21	
> N1	0	0	1	2	0	0		4	4	7	13	
ALND	4	16	14	30	4	25	0.409	55	61	41	79^a^	<0.001
Chemotherapy	4	17	5	11	2	12	0.908	18	20	36	68	<0.001
Endocrine therapy	23	96	40	93	13	81	0.322	70	78	36	86	0.349
Radiotherapy of supra- and infraclavicular nodes	0	0	2	4	0	0	0.693	7	8	10	19	0.049
Medical comorbidities ≥2^b^	14	56	37	80	11	69	0.091	49	54	9	17	<0.001

Abbreviations: TARGIT-A, *TARG*eted *I*ntra-operative radio*T*herapy versus whole breast radiotherapy for breast cancer, IORT, intraoperative radiotherapy, EBRT, external beam whole breast radiotherapy, ALND, axillary lymph node dissection.

* IORT versus EBRT versus IORT-EBRT.

** IORT-EBRT versus IORT-boost versus EBRT-boost.

^a^ One patient without axillary assessment.

^b^ Coexisting medical conditions were assessed by medical record review and by patient self-report. A medical comorbidity was defined as having two or more physical illnesses or injuries that needed long-term treatment.

The present study was performed according to the Declaration of Helsinki principles. All patients were informed about the study and given the option of participating or not. Patients who consented to participate received mailed questionnaires 8 to 94 months following treatment. It was the choice of the patient to answer the QoL questionnaires, and an answer by the patient was considered as informed consent. To maximize response rate, patients were reminded by telephone after non-response.

### QoL measures

Quality of life was assessed using two validated questionnaires of the EORTC: the Quality of Life Questionnaire C30 (QLQ-C30, version 3) and the Breast Cancer Module (QLQ-BR23).

The QLQ-C30 measure consists of one global health status/quality of life scale, five functioning scales (physical, role, emotional, cognitive, and social), three symptom scales (fatigue, nausea/vomiting, and general pain), and six single item scales (dyspnea, insomnia, appetite loss, constipation, diarrhea, and financial impact). The QLQ-BR23 module consists of two functioning scales (body image and sexual), three symptom scales (systemic therapy side effects, breast, and arm symptoms), and three single item scales (sexual enjoyment, future perspective, and upset by hair loss) specific to breast cancer. The scoring of both questionnaires was performed according to the recommended EORTC procedures [6]. All scores can be linearly transformed to a 0–100 point scale, with higher scores of functioning indicating greater functioning, i.e. better QoL, and higher scores on symptoms indicating worse symptoms, i.e. worse QoL. The time frame for all questions is the situation in the last week, except for items related to sexual functioning where a 4-week time frame is taken. Five functioning and symptom scales of the QLQ-C30/QLQ-BR23 questionnaires were preselected during the design of this study, based on a pilot study by our group [7] and relevance for radiation-related QoL in breast cancer: Global health status, restrictions in daily activities (role functioning), and general pain, breast, and arm symptoms. Other subscales and items of the QLQ-C30/QLQ-BR23 questionnaires were outside the aim of this study and are not presented here. The item response rate was between 96% and 99%.

The Hospital Anxiety and Depression Scale (HADS) [8], the Functional Assessment of Cancer Therapy-Fatigue (FACT-F) subscale [9], the Rosenberg Self-Esteem Scale (RSES) [10], and the Body Image Scale (BIS) [11] were used to control for differences that may inherently exist between the treatment groups. The Hospital Anxiety and Depression Scale (HADS) is a questionnaire for the screening of anxiety and depression in patients with physical illness. It consists of two subscales: one for anxiety and one for depression. The Functional Assessment of Cancer Therapy-Fatigue (FACT-F) subscale assesses fatigue and its impact on daily activities. The Rosenberg Self-Esteem Scale (RSES) measures global feelings of self-worth and self-acceptance. The Body Image Scale (BIS) is a valid measure of the affective, cognitive, and behavioral components of body image in cancer patients. The BIS was constructed in collaboration with the EORTC Quality of Life Study Group, and includes the body image subscale of the QLQ-BR23 module.

The scores of the HADS, FACT-F, RSES and BIS questionnaires were summed for each scale. The HADS scores range from 0 (no anxiety/depression) to 21 (severe anxiety/depression). The range of the FACT-F score is from 0 (greatest fatigue) to 52 (lowest fatigue). The RSES score ranges between 10 (low self esteem) and 40 (high self esteem). The BIS score ranges from 0 (best body image) to 30 (worst body image).

### Statistical analysis

Data analyses were performed with the IBM Statistical Package for Social Sciences software, version 19 (SPSS Inc., Chicago, IL). All analyses were performed on an intention-to-treat and as-treated basis. The level of statistical significance was set at 0.01 (0.05/5) to reduce type I errors in multiple comparisons. Chi-squared tests (or Fisher’s exact tests), Kruskal-Wallis one-way analyses of variance, and post-hoc Mann–Whitney U-tests (or univariate analyses of variance and post-hoc Scheffe tests) were used to compare the treatment groups. Independent effects of demographic and clinical factors on QoL were tested using univariate linear regression analysis. Variables with a p value < 0.05 were further analyzed with multiple linear regression analysis (stepwise forward method).

## Results

### Intention-to-treat (ITT) analysis of randomized patients from TARGIT-a trial

In the ITT analyses, patients allocated to IORT showed more professional and other daily activities and fewer general pain symptoms compared to patients allocated to EBRT, but none of the *P* values were below 0.01 (Table 2).

**Table 2:** Preselected QoL variables between TARGIT-A patients allocated to IORT versus EBRT

	**Allocated to IORT**			**Allocated to EBRT**			
**Variable**	**N**^**a**^	**Mean**	**SD**	**N**^**a**^	**Mean**	**SD**	***P***
Global health status^b^	46	61.6	21.7	40	54.8	19.9	0.183
Restrictions in daily activities^b^	46	72.8	32.3	41	61.8	29.2	0.055
General pain^c^	46	29.3	32.8	42	42.5	33.0	0.048
Breast symptoms^c^	45	17.0	20.8	42	18.1	20.2	0.629
Arm symptoms^c^	45	24.4	26.7	40	31.1	27.9	0.279

^a^ Number of valid assessments.

^b^ Higher scores are equal to good functioning/good quality of life.

^c^ Higher scores are equal to severe symptoms/worse quality of life.

### As-treated (AT) analysis of randomized patients from TARGIT-a trial

**Figure 1:**
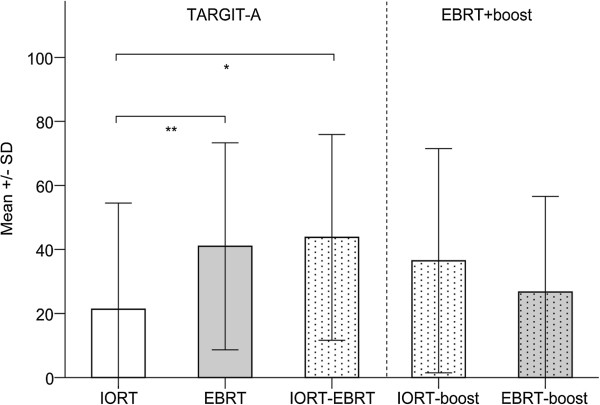
**General pain by treatment group as measured with the EORTC QLQ-C30.** Higher scores are equal to severe symptoms/worse quality of life. Please note: Univariate regression analysis revealed no influence of follow-up duration on self-reported pain symptoms. Abbreviations: SD, standard deviation. * Mann–Whitney U-test. *P* = 0.018. ** Mann–Whitney U-test. *P* = 0.007.

**Figure 2:**
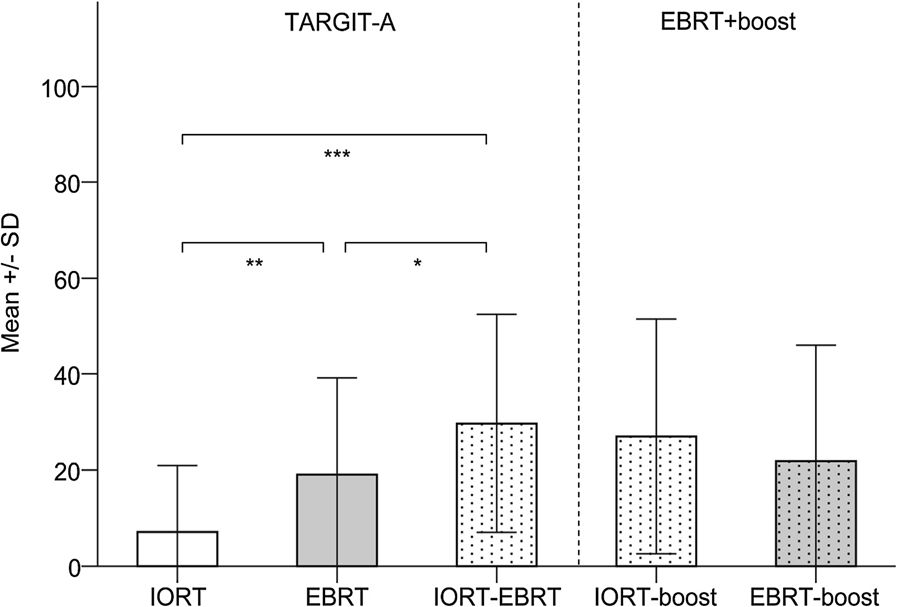
**Breast symptoms by treatment group as measured with the EORTC QLQ-BR23.** Higher scores are equal to severe symptoms/worse quality of life. Please note: Univariate regression analysis revealed no influence of follow-up duration on self-reported breast symptoms. * Mann–Whitney U-test. *P* = 0.021. ** Mann–Whitney U-test. *P* = 0.001. *** Mann–Whitney U-test. *P* < 0.001.

**Figure 3:**
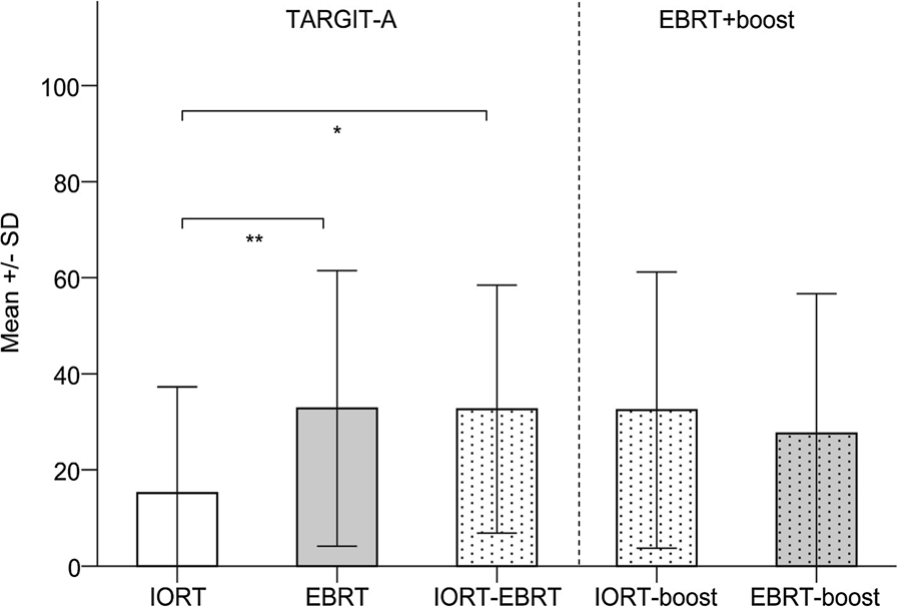
**Arm symptoms by treatment group as measured with the EORTC QLQ-BR23.** Higher scores are equal to severe symptoms/worse quality of life. Please note: Univariate regression analysis revealed no influence of follow-up duration on self-reported arm symptoms. * Mann–Whitney U-test. *P* = 0.011. ** Mann–Whitney U-test. *P* = 0.009.

**Figure 4:**
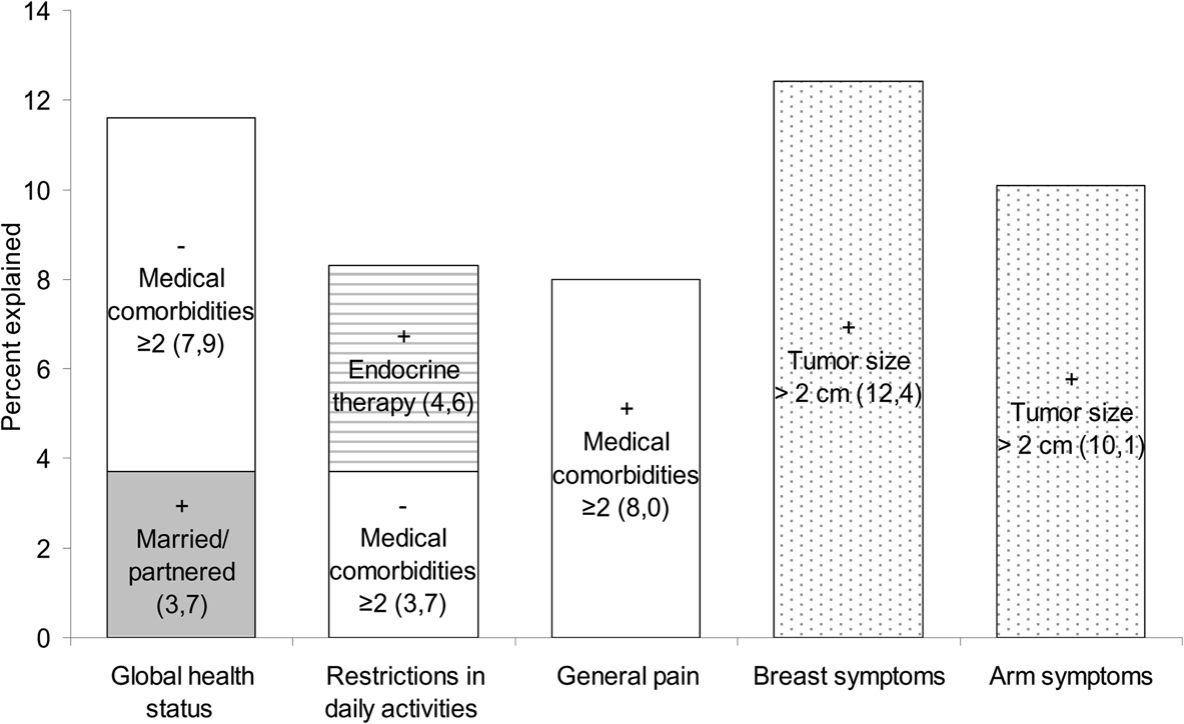
**Percentage of variance in the QoL parameters accounted for by demographic and clinical variables for the TARGIT-A group.** The bars show the percentage of variance explained by multiple linear regression analysis for the QoL parameters examined. The number in brackets indicates the percentage of variance explained by each factor. The sign before each term indicates whether the factor was positively or negatively related to the QoL parameter.

The AT analyses demonstrated a significant benefit for patients treated with IORT alone. Patients receiving IORT alone reported more professional and other daily activities (mean 78.7, SD 35.2) compared to patients receiving EBRT (mean 60.5, SD 29.5; *P* = 0.007). Differences in the global health status subscale were not observed (IORT, mean 63.6, SD 24.2, EBRT, mean 52.4, SD 22.1, IORT-EBRT, mean 60.9, SD 19.9; *P* > 0.01). The mean scores for the general pain, breast, and arm symptom scales were significantly lower in IORT patients than EBRT patients (Figures 1, 2, 3). The difference in breast symptoms between IORT and IORT-EBRT patients was also significant (Figure 2). Between-group differences in the HADS, FACT-F, RSES and BIS scores were not observed (*P* > 0.01). Table 3 summarizes the frequencies of moderate or severe breast and arm symptoms reported by patients. The most commonly reported symptoms were moderate or severe pain in the arm or shoulder, difficulty in raising or moving arm sideways, and pain in the area of the affected breast, with no significant differences between treatment groups (*P* > 0.01). We found only a few associations between demographic and clinical characteristics and QoL parameters. Figure 4 shows the percentage of variance explained by multiple linear regression modeling. Having two or more medical comorbidities was associated with worse global health status, more restrictions in other daily activities, i.e. worse role functioning, and more general pain symptoms (*P* = 0.004 to 0.043). Breast and arm symptoms were independently predicted by tumor size > 2 cm (*P* = 0.003 and 0.002).

**Table 3:** Frequencies of moderate or severe breast and arm symptoms reported by patients in each group (as treated)

	**TARGIT-A**			**EBRT+boost**	
**Symptoms (moderate/severe)**	**IORT (%)**	**EBRT (%)**	**IORT-EBRT (%)**	**IORT-boost (%)**	**EBRT-boost (%)**
Pain in area of affected breast	4/0	11/4	25/13	20/9	11/8
Swelling in area of affected breast	0/0	4/2	7/7	7/10	11/2
Oversensitivity in area of affected breast	4/0	9/7	20/7	20/15	11/8
Skin problems on or in area of affected breast	4/4	9/4	13/6	11/2	4/7
Pain in arm or shoulder	8/8	18/23	33/20	21/15	26/8
Swelling in arm or hand	8/4	9/7	6/6	18/9	19/6
Difficulty in raising or moving arm sideways	20/0	24/12	13/7	16/10	11/8

### Analysis of IORT-EBRT group and Non-randomized comparison groups (IORT-boost and EBRT-boost)

No differences between IORT-EBRT patients and IORT-/EBRT-boost patients were found for any of the QoL comparisons (*P* > 0.01; Figures 1, 2, 3). Table 4 shows the proportion of variance explained in the QoL parameters by each of the demographic and clinical variables. In the multiple linear regression analyses, only one factor was related to 4 out of the 5 QoL parameters: having two or more medical comorbidities was associated with worse global health status, restrictions in daily activities, i.e. worse role functioning, more general pain symptoms, and arm symptoms.

**Table 4:** Proportion of variance explained in the QoL parameters by each of the demographic and clinical variables for the boost groups (IORT-EBRT, IORTboost, and EBRTboost; results from univariate linear regression analyses)

	**Global health status**	**Restrictions in daily activities**	**General pain**	**Breast symptoms**	**Arm symptoms**
Age	3,1%*	2,1%	4,5%*	1%	0,0%
Married/partnered	0,9%	1,6%	1,1%	0,6%	0,3%
Employed	2,4%	3,7%*	4,8%*	0,0%	0,7%
Months since BCS	2,6%*	1,2%	0,3%	1,4%	0,8%
Tumor size	1,3%	0,5%	0,8%	0,2%	0,2%
Nodal involvement	0,6%	0,3%	1,3%	1,3%	0,3%
ALND	1,0%	0,0%	0,0%	0,8%	0,0%
Chemotherapy	3,4%*	3,3%*	4,5%*	0,3%	0,2%
Endocrine therapy	0,0%	0,2%	0,0%	0,7%	0,1%
Radiotherapy of supra- and infraclavicular nodes	1,0%	0,0%	0,2%	0,6%	0,1%
Medical comorbidities ≥2	15,0%**	11,0%**	17,7%**	0,0%	4,9%*

* p < 0,05 in the univariate analysis.

** p < 0,05 in the univariate and multivariate analyses.

## Discussion

The data reported here are the first to examine the effects of IORT on radiation-related QoL parameters in women with breast cancer participating in a randomized trial. Some of our data confirm a previous study by our group reporting on fewer breast symptoms and less general pain symptoms after IORT alone compared to IORT as a tumor bed boost followed by EBRT in breast cancer patients treated outside of the TARGIT-A trial [7]. In the present study, a single-center subgroup of patients of the TARGIT-A trial treated with IORT alone were found to have a significantly better radiation-related QoL than those treated with EBRT as assessed by restrictions in daily activities (role functioning), general pain, breast, and arm symptoms. Patients of the TARGIT-A trial treated with IORT-EBRT had significantly more breast symptoms, and a trend to more general pain and arm symptoms compared to patients receiving IORT alone, but did not differ from patients treated with an IORT boost or EBRT boost outside of the TARGIT-A trial or EBRT without a boost. The most commonly reported symptoms were pain in the arm or shoulder, difficulty in raising or moving arm sideways, and pain in the area of the affected breast. It is important to note that demographic and clinical characteristics had only a minor influence on the QoL parameters assessed here. In addition, there were no further differences between the TARGIT-A or boost groups in the anxiety, depression, fatigue, self-esteem, and body image scores.

Chronic pain in the breast, axilla, or arm is a clinically significant problem in approximately 50% of breast cancer patients [12,13], and a risk factor for poor long-term quality of life [14]. Previously identified risk factors include younger age, adjuvant radiotherapy, and axillary lymph node dissection [12,15]. In our study, general pain symptoms were related to the treatment with IORT (IORT was associated with fewer general pain symptoms) as well as the number of medical comorbidities (having 2 or more medical comorbidities was associated with more general pain symptoms). Neither younger age nor axillary lymph node dissection had a significant effect. Having adjusted for demographic and clinical variables that were found to differ between the treatment groups, only IORT was associated with a reduction in breast and arm symptoms. Consistent with our results, Hopwood *et al.*[13] reported no differences in breast and arm symptoms between patients receiving EBRT with or without an external boost to the tumor bed.

Until now there are only few studies investigating QoL, toxicity and cosmesis after partial breast irradiation. Belkacémi et al. reported a transient decrease in social/family well-being during the first year and an improvement in emotional well-being within the first 24 months after HDR brachytherapy [16]. A superior body image perception was seen after intra- and postoperative interstitial brachytherapy as compared to EBRT [17], and IORT was found to be associated with excellent QoL and few symptoms [18].

The strengths of our study are that it is based on a randomized trial combined with an assessment of various demographic and clinical characteristics, the use of validated disease-specific QoL questionnaires, and the analysis of alternative boost techniques. The main limitation of our study is that it is a cross-sectional study that provided only one estimate of radiation-related QoL variables. Also, the cross-sectional design does not allow drawing conclusions regarding causality, but can describe factors associated with self-reported QoL outcomes. However, our study offers precise estimates regarding self-reported general pain, breast, and arm symptoms and associated factors following radiotherapy with IORT +/− EBRT or EBRT+/− EBRT boost.

## Conclusions

In conclusion, our study is the first in which self-reported QoL symptoms have identified differences between IORT and EBRT. In the randomized setting, important radiation-related QoL parameters after IORT alone were superior to EBRT. IORT patients reported less pain, breast, and arm symptoms, and fewer restrictions in daily activities compared to EBRT patients. IORT-EBRT patients showed a trend to more pain, breast, and arm symptoms compared to IORT alone patients, but did not differ from patients treated with an IORT or EBRT boost outside of the TARGIT trial or EBRT without a boost. In the future, we will extend the sample size and the follow-up time, and we will assess if subjective quality of life scores, objective toxicity scores, and cosmesis are correlated with each other.

## Competing interests

There was no funding of the study. The IORT/TARGIT-A project is supported by grant FKZ 01ZP0508 from the German Ministry for Education and Research (BMBF). Frederik Wenz received funding for radiobiology research from Carl Zeiss Surgical, Oberkochen, Germany. Grit Welzel received funding for neuropsychological research from the Dietmar Hopp foundation. Carl Zeiss sponsors most of the travel and accommodation for meetings of the International Steering Committee and when necessary for conferences where a presentation about targeted intraoperative radiotherapy is being made for all authors.

## Author’s contributions

GW and FW made substantial contributions to the conception and design of the study. GW, AB, ES, FH, UKT, and AG made substantial contributions to the acquisition of data. GW, MS and FW made substantial contributions to the analysis and interpretation of data, and were involved in drafting the manuscript. GW, ES, MS and FW critically revised the manuscript for important intellectual content. All authors read and approved the final manuscript.
